# A Mirror of the Practice of Medicine and Surgery in the Hospitals of London

**Published:** 1871

**Authors:** 


### *A Mirror of the Practice of Medicine and Surgery in the Hospitals of London.*

### KING'S COLLEGE HOSPITAL.

The following operations were performed at this hospital on Saturday, the 6th inst., by Sir Wm. Fergusson:

*Operation for Hare-lip*—This was performed by paring the edges of the gap, sparingly above and freely at the lip, by a stroke of the knife on either side. The edges of the wound having been transfixed by two pins, and secured with a silk suture applied in a figure of 8, the lip was painted with collodion.

Sir Wm. Fergusson expressed his surprise that the old legend, that operations for hare-lip should not be performed until after the completion of the first dentition, should still retain so firm a hold on many minds, in spite of the overwhelming testimony, afforded by modern surgery, of the advantages of operating between the third and sixth weeks of life.

*Question of a Stone in the Ear.*—A child was placed upon the table who had been brought up from the country in order that a stone might be extracted from one of his ears. On introducing a probe, Sir Wm. Fergusson found that it came against a hard substance, which he inclined to think was not a stone, but the bone adjoining the membrana tympani, denuded of periosteum. He therefore had an interview with the child's father, who assured him that a stone had, beyond question, been deliberatly introduced into the child's ear, and added that he himself had seen the stone in the ear. Sir William, therefore, returned into the theatre; but, after again making careful use of the probe, decided to leave the ear alone. He said that the bone about the membrana tympani, which was exceedingly hard, had not unfrequently been mistaken for a stone which persevering attempts were made to extract. A case similar to this one had come before him some time previously, and put him very much on his mettle; but he had decided not to interfere, and he was glad to know that the patient had done well. With regard to the case before him, he was of the opinion that if the object on which the probe impinged were a stone, it would yield a clearer "click," and, when manipulated, would undergo some movement, however slight. The examination he had made left him satisfied that there was no stone in the ear, and he would rather that the patient should incur any risk that non-interference might be supposed to involve than that he should undergo, at his hands, the danger of an attempted extraction.

*Operation for Epithelioma of the Eyelid.*—The patient, a middle-aged man, had an epithelial growth on the surface of the left eyelid. Sir Wm. Fergusson decided to remove the eyelid only, and not to interfere with the globe, though he thought it would be justifiable to remove that also, lest it should become the seat of returning disease. He then raised from the temple a flap of about the size of a half-penny piece, and twisting it on its base, brought it to rest on the eye, and stitched its upper margin to the upper part of the orbital circumference, and to its lower surface a portion of the palpebral mucous membrane which had been preserved.

Sir Wm. Fergusson also operated on a cleft palate, and removed a large sebacious cyst from the loin of another patient.

Mr. Henry Smith re-resected an elbow on which he had performed a partially successful operation some six weeks previously; he found it necessary to remove some portions of dead bone from the extremities of all the three bones which take part in the joints. [*Lancet.*

